# Evaluation of a Brief Online Self-help Program for Concerned Gamblers

**DOI:** 10.1007/s10899-021-10005-6

**Published:** 2021-02-09

**Authors:** Håkan Wall, Kristoffer Magnusson, Anne H. Berman, Bridgette M. Bewick, Clara Hellner, Nitya Jayaram-Lindström, Ingvar Rosendahl

**Affiliations:** 1 Centre for Psychiatry Research, Department of Clinical Neuroscience, Karolinska Institutet, Solna, Sweden; 2 Stockholm Health Care Services, Stockholm County Council, Norra Stationsgatan 69, 11364 Stockholm, Sweden; 3 Department of Psychology, Uppsala University, Uppsala, Sweden; 4 School of Medicine, Division of Psychological and Social Medicine, University of Leeds, Leeds, UK

**Keywords:** Gambling, Self-help, Internet intervention, Gambling helpline

## Abstract

The purpose of this study was to evaluate the feasibility and module content of a brief online self-help program for concerned gamblers, i.e., gamblers who perceived a need to change their gambling habits, in the context of a gambling helpline. The program consisted of four modules based on Motivational Interviewing (MI) and Cognitive Behavioral Therapy (CBT), covering motivation to change, logging gambling behaviors, planning and implementing gambling-free activities, and managing risk situations. Gambling expenditures were also logged in the program, and their development over time were analyzed as longitudinal data using marginalized two-part models. Out of 4655 gamblers recruited via the helpline’s webpage, 92% completed content in at least one module, and 23% were active in all four modules. Attrition was in general high, with only 10% retention in the gambling log for longer than 14 days. Gambling expenditures decreased for those who logged them for a shorter time period, whereas it increased for those who logged expenditures for a longer time period. This study shows that it is relatively easy to recruit participants to an online program for concerned gamblers in the context of a gambling helpline. However, since few users logged in to the program more than once, we suggest future online programs to have open modules with all content accessible at once.

**Supplementary Information:**

The online version contains supplementary material available at(10.1007/s10899-021-10005-6).

## Introduction

The prevalence of problem gambling (PG) is 0.5–3% in different parts of the world (Abbott, Romild, & Volberg 2018) and in Sweden, the current prevalence of PG is estimated at 0.6%, with an additional 3.6% defined as at-risk gamblers (The Public Health Agency of Sweden 2019). PG is associated with serious negative consequences in terms of financial and relational harms and has in the general population been found to coincide with other behavioral addiction problems (Thege, Hodgins, & Wild 2016). Among treatment-seeking individuals with PG, levels of comorbid depression and anxiety are high, and 58% of the treatment-seekers in Sweden were found to have a concurrent psychiatric diagnosis (Håkansson, Mårdhed, & Zaar 2017).

The proportion of individuals with PG who seek help is however low, with an estimate of 7–12%, based on US survey data (Slutske 2006), a level similar to the poor treatment-seeking rates among persons with substance use problems (Kohn, Saxena, Levav, & Saraceno 2004). Shame, stigma and a desire to solve the gambling problem on one’s own, are commonly reported barriers to help-seeking among problem gamblers (PGs) (Gainsbury, Hing, & Suhonen 2014; Suurvali, Cordingley, Hodgins, & Cunningham 2009). Both genders are equally unlikely to seek help, but men express more barriers related to pride and problem denial, compared to women (Rodda, Hing, & Lubman 2014).

Brief interventions have been developed as a low threshold alternative which could be suitable for PGs not seeking treatment. These interventions usually encompass counselling, personalized feedback and/or self-help materials with no guidance at all or minimal therapist support (Swan & Hodgins 2015). Two recent meta-analyses have concluded that these interventions are feasible as harm reduction strategies, at least in the short-term perspective (Peter et al. 2019; Yakovenko, Quigley, Hemmelgarn, Hodgins, & Ronksley 2015). Although there is a growing body of research on brief interventions for PGs, few studies have evaluated interventions for PGs which consist of online self-help, completely without therapist support. In Canada, Hodgins and colleagues tested two modes of delivering relapse prevention tips via email, either as a single email or as seven emails over a one-year period. Both groups spent less money on gambling at the 12-month follow-up, but no difference was found between the groups (Hodgins, Currie, El-Guebaly, & Diskin 2007). In a subsequent study, a brief normative feedback intervention was compared to an extended online intervention consisting of six modules based on a Cognitive Behavioral Therapy (CBT) self-help book for PG. Participants in both groups reduced gambling days and problem gambling severity over time, but no differences were found between the groups. The authors hypothesized that the level of engagement in the extended online intervention would affect the gambling-related outcomes in this group. Indeed, they found that those who engaged in five to six modules gambled fewer days during follow-up compared to those who were less engaged (Hodgins, Cunningham, Murray, & Hagopian 2019). Adding a mental health intervention in conjunction to an online gambling intervention without therapist support did not yield any added benefit, in a study by Cunningham and colleagues (2019). To our knowledge, just one study has evaluated an online intervention for PGs within the context of a gambling helpline. In a study in Australia, gamblers who were already using one or more of the helpline’s e-health services were randomized to either 12 weeks of proactive SMS with self-help tips or to treatment as usual (the helpline’s e-health services). The proactive SMS service consisted of two SMS per week, one containing behavioral change tip and the other SMS was a prompt to give feedback on whether the tip was helpful or not. At the 12-week evaluation both groups had reduced their gambling related outcomes (gambling severity, days gambled and money spent on gambling). However, no differences were found between the groups (Rodda, Dowling, Knaebe, & Lubman 2018). The above-mentioned studies indicate that brief online interventions may be beneficial and that extended programs may not be superior to briefer formats. However, to our knowledge, no study has reported feasibility for an online self-help program for problem gamblers (PGs) in the context of a gambling helpline’s daily operations.

The main objective of the current study was to evaluate the feasibility of a very brief online self-help program for gamblers in the context of a national gambling helpline, as in conversion rate (i.e., the proportion of users who read information about the study and registered an account), program engagement, retention, module content and change in gambling behaviors over time.

## Method

### Design

An uncontrolled open trial with recruitment through the Swedish National Gambling Helpline’s website.

### Procedure and Participants

The Swedish gambling helpline, which was founded in 1999, offers counselling for individuals with gambling problems and their relatives via telephone, chat and email. It is also a knowledge resource for treatment providers. On an annual basis, approx. 1200 individuals with gambling problems, 1000 relatives and 600 treatment providers and other third parties contact the helpline (Stockholm Center for Psychiatry Research 2019). The helpline is funded by the government and has no affiliations with the gambling industry. Previous research on the helpline’s online problem gambling screener indicate that gamblers who come in contact with the helpline via its webpage experience severe gambling problems (Wall, Berman, Jayaram-Lindström, Hellner, & Rosendahl 2020). The helpline also offers an online self-help program without therapist support at its website for gamblers who want to change their gambling habits on their own. The target population for this study were gamblers who registered an account at the online self-help program between 2015–01-28 and 2019–03-19, were 18 years or older and filled in complete background information (gender and game types). This study was approved by the Stockholm regional ethics committee (Dnr: 2017/2210–31/5).

### Intervention

The self-help program was released in March 2015. The program is pure self-help and was developed by the first author as a stand-alone complement to the gambling helpline’s telephone, chat and e-mail services. Gamblers register via the helpline website to get access to the program. The purpose of the program is to provide a set of tools which the users can access, with a goal of promoting behavioral change. During 10 consecutive weeks, participants receive weekly e-mails offering instruction on how to use the program and self-help tips based on well-known techniques from CBT and Motivational Interviewing (MI) (Table 1).

**Table 1:** Themes for weekly e-mails sent to the users for 10 consecutive weeks

Week	Theme
1	Introduction to the program, a prompt to start the gambling log and tips on how to restrict gambling opportunities
2	Motivation to change and tip on how to fill out the motivation module
3	How gambling problems develop and are maintained, tip on how to manage the economy
4	Gambling free activities and tip on how to use the activity planner
5	Risk situations and how to manage them, tip on how to fill out and use the analysis of risk situations module
6	Recap on the work conducted so far
7	Gambling urges and tip on how to continue working with the risk situations module
8	Analyses of gambling behaviors; short- and longtime consequences
9	Lapses and relapses and how to learn from previous relapses
10	Recap on the work conducted during the whole program and tip on how to stay on track

The self-help program consists of four online modules: a motivational balance task, gambling expenditure log, gambling-free activities planner, and analysis of risk situations. All modules are accessible from the user’s personal page, see Table 2 for description of the four online modules. The personal page also has gamification elements and at a set interval, users are awarded virtual badges for working with the modules, as a means to reinforce program engagement. Program users also receive automatized e-mails and SMS prompts as a way to increase program engagement.

**Table 2:** Description of module content

Module	Variables	Input
Motivational balance task Identify pros and cons with changing the gambling habits	Positive and negative aspects of gambling Negative aspects gambling Negative aspects of changing gambling Positive aspects of changing gambling	Pre-defined options available via drop-down lists. Options can also be added via free-text input
Gambling log Set gambling goals Log gambling habits Get visual feedback Get e-mail reminders	*Set weekly gambling goals:*	
	Expenditure	0, 50, 100, 200, 500, 1000, 2000 SEK via drop-down list or via free-text input
	Time	0, 2, 5, 10, 15, 20 h via drop-down list or via free-text input
	*Weekly measures:*	
	Expenditure	0, 50, 100, 200, 500, 1000, 2000 SEK via drop-down list or via free-text input
	Time	0, 2, 5, 10, 15, 20 h via drop-down list or via free-text input
	Cravings	VAS scale 0–4, with 0 representing “none” and 4 representing “a lot”
	Well-being	VAS scale 0–4, with 0 representing “bad” and 4 representing “good”
Gambling free activities planner Plan activities and get e-mail reminders	Gambling free activities	Activities are added as free-text input, time and date set in a calendar-type view
Risk situations Identify risk situations and strategies to manage them Chose strategy in “hot state” E-mail reminder to evaluate strategy	Risk situations and strategies. Several strategies can be added per risk situation	Pre-defined options available via drop-down lists for risk situations and strategies. Options can also be added via free-text

The self-help program consists of four online modules: a motivational balance task, gambling expenditure log, gambling-free activities planner, and analysis of risk situations. All modules are accessible from the user’s personal page, see Table 2 for description of the four online modules. The personal page also has gamification elements and at a set interval, users are awarded virtual badges for working with the modules, as a means to reinforce program engagement. Program users also receive automatized e-mails and SMS prompts as a way to increase program engagement.

### Statistical Method

Free-text input from the modules was analyzed using the R-package tidytext (Silge & Robinson 2016). All free-text data was compiled into a corpus where each entry (a sentence or a single word) was divided into single words; i.e., the corpus contained a module column and a word column, for instance the sentence “I lose money”, resulted in three rows: “I”, “lose” and “money”. Based on term frequency (tf; i.e., how often the term occurred) and the inverse document frequency (idf = ln(n_documets_/n_documents containing the term_)), the product tf-idf was calculated for each word, which indicated which words are important in each module (Silge & Robinson 2017). The 10 words with the greatest tf-idf weights in each module were considered significant keywords for that module. Finally, to validate the keywords, we calculated the proportion of entries in each module that contained the keywords. Keywords that shared more than 10% of entries were removed, for instance “lose” and “money” co-occur in “lose money”, if the keyword with least weight, in this example “money”, occurs in more than 10% of the entries for the keyword with the greater weight, in this case “lose”, the keyword with least weight, “money”, will be removed and replaced with the next keyword in line. The 10 keywords with the greatest weights in each module, after the external validation, and example sentences for each key-word are presented.

The overall gambling expenditures were analyzed using a longitudinal marginalized two-part model. This model is flexible enough to account for the large number of reports of zero losses, while also allowing for the fact that gambling losses are typically heavily right skewed (Magnusson, Nilsson, & Carlbring 2019). This is achieved by combining two generalized linear mixed-effects models (GLMM) – a logistic GLMM for the zero part and a skewed continuous (G)LMM for the nonzero expenditure (Magnusson et al. 2019; Smith, Neelon, Preisser, & Maciejewski 2017). Since we are interested in the overall reduction in expenditure the model is reparametrized so that change in expenditure refers to the overall expenditure including zeros. We modelled change over time as a continuous linear variable; however, due to the widely varying “duration of logging” we let the users’ slopes vary as a function of duration of days when they used the log. Days of logging were modelled using natural cubic spline with three degrees of freedom. The R-package brms (Bürkner 2017) which is a higher-level interface for the probabilistic programming language Stan (Carpenter et al. 2017) together with a custom brms family for a marginalized two-part lognormal was used to fit the model (see Magnusson et al. 2019).

## Results

Out of 40,256 individuals who read information about the program on the website, 8083 visited the program registration page and 5652 registered an account, which gives a conversion rate of 14%. Most users (n = 4,708) provided complete background information and of these 4655 were included in the study. A majority were male, 67.3%, and 66.5% were aged between 25 and 44 years. Online casino games constituted the most common game type (78.8%) followed by online sports betting (25.9%) and EMG (17.1%). See Table 3 for demographic and gambling characteristics.

**Table 3:** Participant demographic and baseline gambling characteristics

Variable	N = 4,655
*Demographic characteristics*	
Males, n (%)	3165 (67.3)
*Age groups, n (%)*	
18–24	777 (16.5)
25–34	1949 (41.4)
35–44	1125 (24.0)
45–54	552 (11.7)
55–64	210 (4.5)
> 64	42 (0.9)
*Game types (%)*	
Online casino	78.8
Online sports betting	25.9
EGM	17.1
Online poker	12.7
Land-based sports betting	12.3
Horse betting	11.0
Lotteries	7.7
Land-based casino games	7.0
Other types of gambling	6.2
Bingo	5.3
Land-based poker	4.4
Keno-type games	3.4
*Number of games played*	
Mean (SD)	1.92 (1.59)
Median (IQR)	1 (1)

### Program Engagement

Of 4655 registered users, 4273 (91.8%) engaged in the program, i.e., filled out some content in at least one module. The motivational balance task was the most popular module (88.2%) and the gambling expenditure log the least popular (35.1%). The most common patterns of engagement were being engaged in three (30.8%), two (24.6%) or four (23.5%) modules. The most common combination of modules was being engaged in all four modules (23.5%).

### Motivational Balance Module

The positive aspects of changing the gambling habits had the most entries, mean = 3.79 (SD = 2.2), followed by negative aspects of continuing gambling, 3.18 (1.83), negative aspects of changing the gambling habits, 1.81 (1.07), and positive aspects of continuing gambling, 1.78 (1.02). Table 4 shows the proportions of pre-defined aspects chosen in each part of the module. See Tables 5–11 in the supplementary materials for descriptive statistics and example sentences for all content added via free-text.

**Table 4:** Pre-defined reasons chosen in the decision balance module

Decision balance module	n (%)
*Positive aspects of continuing gambling*	4114 (100)
Winning money	2802 (69.8)
Excitement	1693 (42.2)
The kick	1592 (39.7)
Being part of something	218 (5.4)
*Negative aspects of continuing gambling*	4077 (100)
Anxiety	3106 (76.3)
not having enough money	2842 (69.8)
Debts	2463 (60.5)
Sleeping bad	1192 (48.9)
Arguing with those close to me	1491 (36.6)
*Negative aspects of changing gambling habits*	3934 (100)
Not being able to win	1979 (50.3)
Abstinence	1551 (39.5)
Missing the kick	1493 (38.0)
Nothing to do	1343 (34.2)
*Positive aspects of changing the gambling habits*	4091 (100)
Feeling better	3175 (77.6)
Having money to do other things	3110 (76.0)
Less stress	2534 (61.9)
Better health	2164 (52.9)
More time for the family	1779 (43.5)
Being a hands-on parent	2164 (52.9)
Meeting with friends again	1053 (25.7)

### Gambling Log

**Fig. 1:**
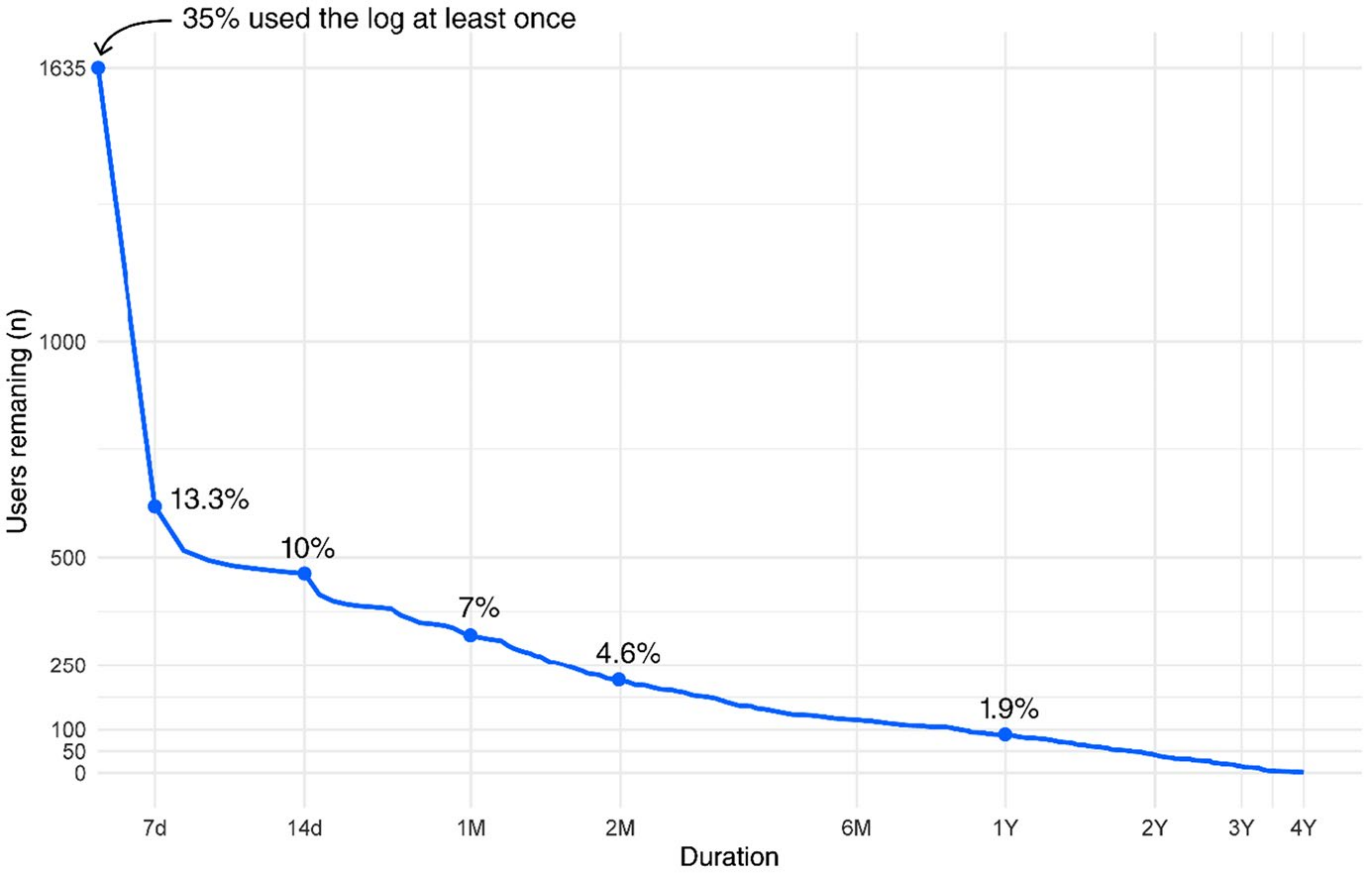
Length of log usage (baseline to last log time point). The x-axis is shown on the log scale

**Fig. 2:**
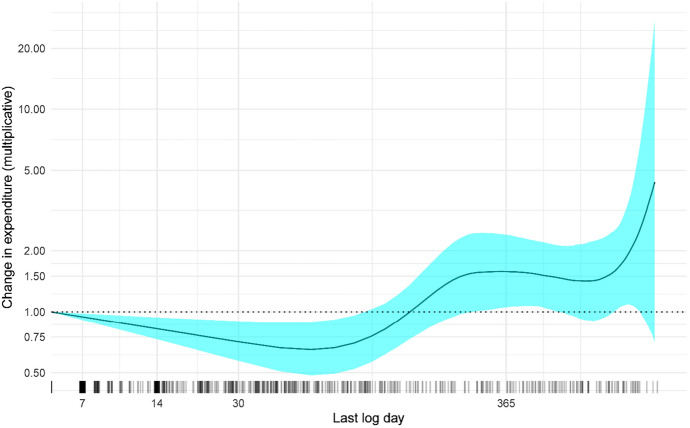
Change in gambling expenditure as a function of the duration of log usage. The y-axis shows the ratio of the expenditure at the last log over the pretest expenditure, values less then 1 indicate a reduction in gambling expenditure. Both axes are shown on the log scale. The rug below the x-axis show the individual data points

Almost a third of the users, 1635 (31.5%), were active in the gambling log. The median spending goal was set to 100 (500) SEK and 40.7% stated a spending goal of 0 SEK. The median time goal was set to 2 (5) hours and 46.4% stated a time goal of 0 h. The median expenditure at the first log occasion was 1000 (200) SEK and the median time gambling was 5 (15) hours. Most users visited the log only once, and l0% of the users remained in the log after 14 days, see Fig. 1 for retention in the gambling log. Figure 2 shows that users who only used to log for a short period (less than three months) reported a decrease of around 20 to 35% in gambling expenditure compared to their first logged expenditure. However, users who used the log for longer periods tended to report an increase in expenditure as compared to their baseline expenditure. Although those who used the log a longer time period increased gambling expenditures, both the proportion of users who reached their gambling goal increased from the first to the last log, from 48 to 56% (McNemar's χ2 = 12.69, *p* = 0.0004), as did the proportion of users reporting 0 SEK expenditure, from 41 to 46% (McNemar's χ2 = 6.5, *p* = 0.01).

### Gambling-Free Activities

Almost half of the users, 2052 (44%), were active in the gambling free activities module. The median number of planned activities were 1 (1) and median time between the first and last activity was 0 (IQR = 2) days. Most users, 66.8%, planned gambling-free activities on just one occasion and 12.3% planned activities that were to be due on at least seven days apart. Among the gambling-free activities, the 10 keywords with the greatest weights were: *walk, gym, call, exercise, tidy/clean, SVT-play (the Swedish public national TV-streaming service), film, cook, meet (as in meet with friends),* and *significant other*. They were represented in 41.8% of all entries, see table 9 in the supplementary material for more details. Further, out of 4,011 planned activities, 385 users marked 669 activities as “done”, i.e., the user ticked the “done box” for the activity.

### Risk Situations

This module was the second most popular, 3627 (77.9%) users added at least one risk situation and 3520 (75.6%) users added a risk situation with an associated coping strategy. Among the pre-defined risk situations, the most frequently endorsed risk situations with corresponding coping strategies were: *getting paid* and *transferring money to someone close to you* (n = 1316, 37.4%), being *home alone* and *going for a walk* (n = 1281, 36.4%), and *stressed* and *going for a walk* (n = 710, 20.2%). About a quarter of the module users, n = 835, used the module in “hot state”, i.e., chose a coping strategy based on a risk situation and were reminded via email or SMS to evaluate it. Among these users, the median number of “hot state” activations were 2 (3) and the median time between the first and last “hot state” activation was 0 (7) days. Further, 213 module users evaluated if the strategy had been successful or not. The risk situation *home alone* and the coping strategy *going for a walk* had most evaluations, it was evaluated as successful on 76 occasions and unsuccessful on 18, *stressed* and *going for a walk* was evaluated as successful on 55 occasions and unsuccessful on 17 and *getting paid* and *going for a walk* was evaluated as successful on 42 occasions and unsuccessful on 14.

## Discussion

In this study, we evaluated the feasibility of a brief (four-module) online self-help program for gamblers accessing the Swedish national gambling helpline website. The conversion rate was 14% in the current study, which can be considered as high compared to a feasibility study on a program for persons affected by someone else’s gambling where the conversion rate was 3.5% (Buchner, Koytek, Wodarz, & Wolstein 2019). Program engagement was high, with 92% engaged in at least one module and 23.5% of the users having been engaged in all four modules. In the only comparable study targeting PGs by Hodgins and colleagues, 57% of the users accessed the program and 31% of the users accessed all modules (Hodgins et al. 2019). In Buchner et al. (2019), who targeted persons affected by someone else’s gambling, two thirds accessed the program, and 37% engaged in all five modules. These differences in program engagement could be due to differing program set-ups. In Hodgins et al. (2019) users were prompted to engage in the modules which fitted their needs while in Bucher et al. (2019) modules were offered once per week. In the current study all modules were accessible at the first visit. Further, program engagement may depend on if the users are motivated to change the target behavior or not. In a smoking cessation program, 37% never logged in at all, and a majority of those users were not interested in changing their smoking habits or intended to stop smoking at a later time point (McClure et al. 2013). Probably, many users in the current study registered an account just to get a glimpse of what the program was about without intention of changing their gambling behaviors.

Furthermore, few users returned to the program over time, most just visited the program once and a small proportion of users are active in the program for a longer time period. Since data on actual logins was not registered, retention time can only be estimated via the modules where time stamps were used, i.e., in the gambling-free activities module, the gambling expenditure log and the relapse prevention module. In the gambling log, the median retention time (i.e., time to the last log occasion) was 0 (IQR = 15) days for all users active in the log, and among those who used the log on at least two occasions (n = 618) the median retention time was 33.5 (93.2) days. In the gambling-free activities module the time between the first and last planned activity can be viewed as a proxy for retention time. However, a user could add multiple activities over a long time period without ever visiting the program again. Given these conditions, the median time between first and last planned activity was 0 (2) days for all users active in this module and 4 (17.2) days for those who planned activities with at least one day apart. In the relapse prevention module, the recorded time stamp represents an actual login to the system, and the median time between the first and last usage of the module was 0 (7) days for all who engaged in the module and 8 (30) days for those activated a reminder on more than one occasion. Similar results were found in in study on an activity tracker (Guertler, Vandelanotte, Kirwan, & Duncan 2015) where the median retention time was 30 days, and in a study on a web-based self-help program targeting problematic alcohol use where most users visited the program on one to two occasions (Johansson et al. 2016). This information is relevant to consider when developing programs, as expecting users to return to the program on a regular basis for a longer time period appears unrealistic in this type of specific populations.

A better strategy may be to provide all the material at once, instead of portioning out the material over time. The use of e-mail prompts may not be optimal, as not all service providers will identify the e-mails as trusted and may end up as “spam” or be sent directly to the trash can. Building programs as native applications (apps) maybe a better solution for several reasons: easier access for the users, prompts can be sent without having to go through an e-mail or a SMS provider, and makes it easier to control the behavior of, for instance, interactive elements. However, the relatively high development and maintenance costs associated with native apps maybe curbing the extent to which program developers can realistically use native app platforms.

Previous studies on natural recovery from PG and on self-help online interventions for PGs have shown that problem gambling severity, gambling losses and time spent gambling decrease over time (e.g., Hodgins & El-Guebaly 2000; Kushnir et al. 2018).

This study was a naturalistic study with no additional research-related requirements from participants, usually, joining a study demands more from the study participants and this is likely to affect outcomes. For example, Kushnir et al. (2018) highlight that although participants in their study did not receive an intervention per se, several participants displayed their gratitude and left testimonials on how the study had helped them overcome their gambling problems. In the current study the difference in weekly gambling expenditure between baseline and the one-month timepoint was approximately 13 EUR for those who remained in the program for a month. At the 4- week timepoint fewer than 10% remained in the program, which means that for most users the weekly expenditure decreased or remained at approximately the same level.

### Strengths and Weaknesses

High ecological validity is a major strength of the current study, since it includes a naturalistic sample of users in a self-help intervention program for gamblers. Some limitations also deserve mention. First, no control group was used, so no conclusions can be drawn regarding the effectiveness of the intervention. Secondly, PG severity levels were not measured, however, from previous research we know that the gamblers who are in contact with the helpline experience severe gambling problems, and it is likely that the participants in this study were individuals with gambling problems. Thirdly, we do not know if the participants in this study were gamblers, relatives or third party, although it is unlikely that other types of participants than gamblers would use the program to a greater extent. Consequently, this uncertainty may add bias to the results in this study. Fourthly, there was a lack of data on retention measures such as the number of logins or time spent in the program. However, the results give valuable insights on how users engage in a self-help program for concerned gamblers and how their gambling behaviors develop over time.

## Conclusion

The current study shows that it is relatively easy to recruit participants to a self-help online program for concerned gamblers within the context of a gambling helpline. However, few users return to the program over time. Given that most users remained in the program for a short period of time, we suggest that future self-help online programs should have open modules, with all information accessible at once. In order to increase retention, we suggest that programs are developed in close cooperation with its future users, namely the gamblers. Finally, more research is warranted, both on feasibility and the effectiveness of brief self-directed online interventions for PGs in an ecologically valid setting.

## Supplementary Information

 Supplementary file 1 (DOCX 27 kb)
