# Supplementary material for: Evaluation of a Brief Online Self-help Program for Concerned Gamblers
Source: J Gambl Stud. 2021 Feb 9;37(4):1277–90. doi: 10.1007/s10899-021-10005-6 (PMC8572834; doi:10.1007/s10899-021-10005-6)
Supplement: Supplementary file 1 — (DOCX 27 kb) [file 10899_2021_10005_MOESM1_ESM.docx]

# Supplementary material

Table 5. Keywords for positive aspects concerning continued gambling added via free text responses. The keywords were represented in 22.9 % of the free-text entries.

| **Keyword** | **n** | **Total** | **Tf_idf** | **Example sentences** |
| --- | --- | --- | --- | --- |
| Nothing | 70 | 2,741 | 0.0095279 | ”nothing”, ”absolutely nothing” |
| Moment | 9 | 2,741 | 0.0070846 | ”reduces anxiety in the moment”, “the joy of the moment” |
| Killing time | 20 | 2,741 | 0.0068552 | ”killing time”, ”a way to kill time” |
| Escape | 10 | 2,741 | 0.0050678 | ”an escape from reality”, ”the escape” |
| Forget | 8 | 2,741 | 0.0040542 | ”forget the present moment”, ”forget my life” |
| Fun | 16 | 2,741 | 0.0036221 | ”fun when winning”, ”fun when things are going well” |
| Relaxation | 9 | 2,741 | 0.0030848 | ”relaxation” |
| Reason | 7 | 2,741 | 0.0023993 | ”there are no good reasons” |
| Annoying | 7 | 2,741 | 0.0023993 | ”get rid of annoying thoughts” |
| Cure | 3 | 2,741 | 0.0023615 | ”a cure for boredom” |

*Note. Tf_idf refers to the product of term frequency x inverse document frequency, which indicate which keywords are significant in a specific module.*

Table 6. Keywords for negative aspects concerning continued gambling added via free text responses. The keywords were represented in 11.8 % of the free-text entries.

| **Keyword** | **Nn** | **Total** | **Tf_idf** | **Example sentences** |
| --- | --- | --- | --- | --- |
| Destroy | 26 | 4,034 | 0.0082439 | ”I destroy my life”, ”I destroy my relationships” |
| Let down | 15 | 4,034 | 0.0073877 | ”I let down my family”, “I let down those close to me” |
| Lose | 12 | 4,034 | 0.0059101 | ”I lose focus”, ”lose joy” |
| Suicide thoughts | 11 | 4,034 | 0.0054176 | ”suicide thoughts” |
| Conscience | 13 | 4,034 | 0.0041220 | “guilty conscience” |
| Disappoint | 8 | 4,034 | 0.0039401 | “disappoint myself”, “disappoint those close to me” |
| Neglect | 7 | 4,034 | 0.0034476 | “neglect the children”, “neglect the fun of life” |
| Risk | 7 | 4,034 | 0.0034476 | “risk my relationships”, “risk the economy” |
| Lie | 10 | 4,034 | 0.0031707 | “have to lie”, “all lies” |
| Feelings of guilt | 10 | 4,034 | 0.0031707 | “feelings of guilt” |

*Note. Tf_idf refers to the product of term frequency x inverse document frequency, which indicate which keywords are significant in a specific module.*

Table 7. Keywords for negative aspects concerning changed gambling habits added via free text responses. The keywords were represented in 40.2% of the free-text entries.

| **Keyword** | **n** | **Total** | **Tf_idf** | **Example sentences** |
| --- | --- | --- | --- | --- |
| Miss | 21 | 2,189 | 0.0110563 | “miss the feeling of being someone”, “miss the excitement”, ”miss the kick” |
| Bad | 93 | 2,189 | 0.0070068 | ”there is nothing bad”, ”nothing bad about changing” |
| Nothing | 34 | 2,189 | 0.0055615 | ”nothing” |
| Change | 14 | 2,189 | 0.0052518 | ”have to change”, ”good to change” |
| Chance | 16 | 2189 | 0.0034687 | ”no chance of winning”, ”the chance to win extra money disappears” |
| Restless | 16 | 2,189 | 0.0031796 | “restless”, ”will become restless and annoyed” |
| Negative | 6 | 2,189 | 0.0021883 | ”no negative aspects of quitting” |
| Stop/quit | 7 | 2,189 | 0.0020234 | ”only positive to stop ”, ”things can get better if I quit” |
| Income | 2 | 2,189 | 0.0020102 | ”no extra income” |
| Remain | 2 | 2,189 | 0.0020102 | ”the thoughts of winning remain” |

*Note. Tf_idf refers to the product of term frequency x inverse document frequency, which indicate which keywords are significant in a specific module.*

Table 8. Keywords for positive aspects concerning changed gambling habits added via free text responses. The keywords were represented in 22.0 % of the free-text entries.

| **Keyword** | **n** | **Total** | **Tf_idf** | **Example sentences** |
| --- | --- | --- | --- | --- |
| Lie | 23 | 3,811 | 0.0081005 | ”no need to lie anymore”, “can stop lying” |
| Happier | 19 | 3,811 | 0.0066917 | ”happier”, ”will become happier” |
| Better | 89 | 3,811 | 0.0038570 | ”better mood”, ”better economy” |
| Present | 13 | 3,811 | 0.0030967 | ”can be more in the present”, ”be a present partner” |
| Relationship | 8 | 3,811 | 0.0028176 | ”better relationships”, ”better relationship with my family” |
| Partner | 5 | 3,811 | 0.0027353 | ”become a better partner”, ”being able to meet a new partner” |
| Safety | 5 | 3,811 | 0.0027353 | ”safety for my family” |
| Conscience | 7 | 3,811 | 0.0024654 | ”avoid bad conscience” |
| Trust | 4 | 3,811 | 0.0021883 | ”the ones I love can trust me again” |
| Calmer | 4 | 3,811 | 0.0021883 | ”become calmer” |

*Note. Tf_idf refers to the product of term frequency x inverse document frequency, which indicate which keywords are significant in a specific module.*

Table 9. Keywords for gambling free activities added via free-text. The keywords were represented in 41.8% of the free-text entries.

| **Keyword** | **n** | **Total** | **Tf_idf** | **Example sentences** |
| --- | --- | --- | --- | --- |
| Walk | 413 | 10,960 | 0.0552532 | “go for a walk”, “take a walk” |
| Gym | 246 | 10,960 | 0.0329111 | “go to the gym”, “the gym” |
| Call | 119 | 10,960 | 0.0159204 | “call the helpline”, “call a friend” |
| Exercise | 252 | 10,960 | 0.0150601 | “do exercise”, “exercise my dogs” |
| Tidy/clean | 91 | 10,960 | 0.0121744 | “tidy at home”, “tidy the flat” |
| SVT-play | 46 | 10,960 | 0.0095591 | “watch SVT-play” |
| Film | 54 | 10,960 | 0.0072244 | “watch a film” |
| Cook | 44 | 10,960 | 0.0058865 | “cook” |
| Meet | 64 | 10,960 | 0.0057910 | “meet with friends”, “meet with a specific person” |
| Significant other | 91 | 10,960 | 0.0054384 | “transfer money to a significant other”, “take a walk with a significant other” |

*Note. Tf_idf refers to the product of term frequency x inverse document frequency, which indicate which keywords are significant in a specific module. SVT-Play is the national public service television streaming service.*

Table 10. Keywords for risk situations added via free-text responses. The keywords were represented in 38.1% of the free-text entries.

| **Keyword** | **n** | **Total** | **Tf_idf** | **Example sentences** |
| --- | --- | --- | --- | --- |
| Bored | 131 | 3,538 | 0.0220679 | “when I am bored”, “bored at night” |
| Alcohol | 57 | 3,538 | 0.0214953 | “alcohol”, “too much alcohol” |
| Advertising | 47 | 3,538 | 0.0177242 | “advertising on tv”, “advertising from gambling companies” |
| Restless | 73 | 3,538 | 0.0122974 | “restless” |
| Pain | 7 | 3,538 | 0.0041004 | “when I am in pain” |
| Boredom | 15 | 3,538 | 0.0038258 | “boredom” |
| Depressed | 14 | 3,538 | 0.0035708 | “when I feel depressed”, “depressed and low of energy” |
| Paid | 6 | 3,538 | 0.0035146 | “when I have paid the bills and have money left” |
| Work | 34 | 3,538 | 0.0034437 | “bored at work”, “after work” |
| Browse the internet | 9 | 3,538 | 0.0033940 | “when I browse the internet with my phone” |

*Note. Tf_idf refers to the product of term frequency x inverse document frequency, which indicate which keywords are significant in a specific module.*

Table 11. Keywords for coping strategies added via free-text responses. The keywords were represented in 29.1% of the free-text entries.

| **Keyword** | | **n** | **Total** | **Tf_idf** | **Example sentences** |
| --- | --- | --- | --- | --- | --- |
| Call | 72 | | 9,699 | 0.0100266 | “call my mother”, “call home” |
| Film | 52 | | 9,699 | 0.0072414 | “watch a film” |
| Talk | 103 | | 9,699 | 0.0064073 | “talk to a friend”, “talk to my wife” |
| Think | 55 | | 9,699 | 0.0051802 | “think about the future”, “think of the family” |
| Savings account | 30 | | 9,699 | 0.0041777 | “transfer money to a savings account” |
| Music | 24 | | 9,699 | 0.0033422 | “listen to music”, “create music” |
| Read | 23 | | 9,699 | 0.0032029 | “read a book”, “read a newspaper” |
| Tidy/clean | 18 | | 9,699 | 0.0025066 | “tidy at home”, “clean the car” |
| Buy | 17 | | 9,699 | 0.0023674 | “buy something nice”, “go and buy coffee” |
| Shop | 15 | | 9,699 | 0.0020889 | “shop food”, “shop something I need” |

*Note. Tf_idf refers to the product of term frequency x inverse document frequency, which indicate which keywords are significant in a specific module.*
